# Imaging *in situ *breast carcinoma (with or without an invasive component) with technetium-99m pentavalent dimercaptosuccinic acid and technetium-99m 2-methoxy isobutyl isonitrile scintimammography

**DOI:** 10.1186/bcr948

**Published:** 2004-11-08

**Authors:** Vassilios Papantoniou, Spyridon Tsiouris, Ekaterini Mainta, Varvara Valotassiou, Michael Souvatzoglou, Maria Sotiropoulou, Lydia Nakopoulou, Dimitrios Lazaris, Androniki Louvrou, Maria Melissinou, Artemis Tzannetaki, Ioannis Pirmettis, John Koutsikos, Cherry Zerva

**Affiliations:** 1 Department of Nuclear Medicine, 'Alexandra' University Hospital, Athens, Greece; 2 Department of Pathology, 'Alexandra' University Hospital, Athens, Greece; 3 Department of Pathology, University of Athens School of Medicine, Athens, Greece; 4 Department of Obstetrics and Gynecology, 'Alexandra' University Hospital, Athens, Greece; 5 Department of Internal Medicine, 'Metropolitan' Hospital, Athens, Greece; 6 Department of Radiology, 'Alexandra' University Hospital, Athens, Greece; 7 Institute of Radioisotopes – Radiodiagnostic Products, National Center for Scientific Research 'Demokritos', Athens, Greece

**Keywords:** ductal carcinoma *in situ*, extensive intraductal carcinoma, lobular breast carcinoma *in situ*, scintimammography, ^99m^Tc-(V)DMSA, ^99m^Tc-Sestamibi

## Abstract

**Introduction:**

The aim of the study was to retrospectively define specific features of the technetium-99m pentavalent dimercaptosuccinic acid (^99m^Tc-(V)DMSA) and technetium-99m 2-methoxy isobutyl isonitrile (^99m^Tc-Sestamibi [^99m^Tc-MIBI]) distribution in ductal breast carcinoma *in situ *and lobular breast carcinoma *in situ *(DCIS/LCIS), in relation to mammographic, histological and immunohistochemical parameters.

**Materials and methods:**

One hundred and two patients with suspicious palpation or mammographic findings were submitted preoperatively to scintimammography (a total of 72 patients with ^99m^Tc-(V)DMSA and a total of 75 patients with ^99m^Tc-Sestamibi, 45 patients receiving both radiotracers). Images were acquired at 10 min and 60 min, and were evaluated for a pattern of diffuse radiotracer accumulation. The tumor-to-background ratios were correlated (T-pair test) with mammographic, histological and immunohistochemical characteristics.

**Results:**

Histology confirmed malignancy in 46/102 patients: 20/46 patients had DCIS/LCIS, with or without coexistent invasive lesions, and 26/46 patients had isolated invasive carcinomas. Diffuse ^99m^Tc-(V)DMSA accumulation was noticed in 18/19 cases and ^99m^Tc-Sestamibi in 6/13 DCIS/LCIS cases. Epithelial hyperplasia demonstrated a similar accumulation pattern. The sensitivity, specificity, accuracy, positive predictive value and negative predictive value for each tracer were calculated. Solely for ^99m^Tc-(V)DMSA, the tumor-to-background ratio was significantly higher at 60 min than at 10 min and the diffuse uptake was significantly associated with suspicious microcalcifications, with the cell proliferation index ≥ 40% and with c-*erbB-2 *≥ 10%.

**Conclusion:**

^99m^Tc-(V)DMSA showed high sensitivity and ^99m^Tc-Sestamibi showed high specificity in detecting *in situ *breast carcinoma (^99m^Tc-(V)DMSA especially in cases with increased cell proliferation), and these radiotracers could provide clinicians with preoperative information not always obtainable by mammography.

## Introduction

Ductal carcinoma *in situ *(DCIS) of the breast, alone or in the presence of an invasive tumor (invasive ductal carcinoma [IDC]), presents a problem in its detection and treatment. DCIS represents a number of biologically different processes that exhibit variable frequencies of occult invasion and variable risks for local recurrence, after attempts at excision biopsy or lumpectomy. The term 'extensive intraductal component' or 'extensive intraductal carcinoma' (EIC) is defined as DCIS within and around an invasive tumor, comprising at least 25% of the neoplasm. Of all IDC cases, 15–30% are considered to have EIC [1]. Tumors that are predominantly DCIS and appear with focal invasion are also classified as EIC.

DCIS not within the confines of the invasive tumor, but frequently in the adjacent breast tissue, makes breast-conserving surgical treatment less safe with regard to the recurrence rate. The great majority of recurrences are observed at or near the site of the primary tumor. On pathological and radiological evaluation of mastectomy specimens, some studies [1] have shown that EIC tumors commonly had DCIS not only in their immediate vicinity, but also as far as 4–6 cm from the invasive tumor. The risk of local recurrence after a breast-conserving procedure can be evaluated according to the histological type, the extent of the lesion and the state of the surgical margins. It has been reported that the local recurrence rate after a limited resection of the tumor, followed by radiotherapy, is significantly greater for EIC-positive patients than for EIC-negative patients [2,3]. If the real extent of EIC was known preoperatively, it might be possible to safely limit the breast resection, since an initial wide excision would constitute overtreatment for the majority of patients not having a substantial amount of DCIS outside the invasive tumor. The fact that younger patients are more likely than older ones to have EIC, and that the association between EIC and breast cancer recurrence is stronger in premenopausal women than in postmenopausal women, renders the detection of such lesions imperative [1].

Mammographic evidence of microcalcifications with a certain form and distribution is presently the only method of detecting the presence of DCIS. The failure of mammography to recognize DCIS or EIC in radiologically dense breasts in young populations raises a significant problem.

Scintimammography with technetium-99m 2-methoxy isobutyl isonitrile (^99m^Tc-Sestamibi [^99m^Tc-MIBI]) has been used to detect primary breast cancer [4-8]. It concentrates in cancer cells by an energy-requiring transport mechanism and a transmembrane electronegative potential, in addition to nonspecific mechanisms, and it is stored within the mitochondria. Only a few reports [9,10] have discussed the efficacy of ^99m^Tc-Sestamibi in detecting DCIS.

Technetium-99m pentavalent dimercaptosuccinic acid (^99m^Tc-(V)DMSA) is a tumor-seeking agent already known for its efficacy in detecting medullary thyroid carcinoma. Its mechanism of accumulation has been thought to be related to the structural similarity between the (V)DMSA core and the phosphate anion (PO_4 _^3-^), which is avidly taken up by some cancer cells [11,12]. Denoyer and colleagues recently reported that the radiotracer uptake is specifically mediated by NaPi cotransporter type III in cancer cells, similar to phosphate ions, which enter cells via NaPi cotransporters [13], while other reports suggest that its uptake in tumors is related to glucose metabolism-mediated acidosis [14]. ^99m^Tc-(V)DMSA has been tried out in identifying invasive and preinvasive breast cancer [15-17].

The present study retrospectively assessed the capability of ^99m^Tc-(V)DMSA and ^99m^Tc-Sestamibi to image DCIS and EIC. The study also investigated whether this was related to the presence of suspicious microcalcifications on mammography, stromal reaction, lymphocytic infiltration and immunohistochemical parameters such as the cell proliferation index (Ki-67), c-*erbB-2*, and *p53 *expression.

## Materials and methods

### Patients and scintimammography

A total of 102 women (mean age ± standard deviation [SD], 62.5 ± 12.49 years) referred with suspicious breast lesions on physical examination and/or an abnormal mammogram underwent ^99m^Tc-Sestamibi and/or ^99m^Tc-(V)DMSA scintimammography prior to any surgical intervention. All patients were intended to have studies performed with both agents. The first agent to be used was selected on a random basis. Not all patients managed to have the second study performed preoperatively, for various reasons. Forty-five patients underwent both ^99m^Tc-(V)DMSA and ^99m^Tc-Sestamibi breast scintigraphy at separate sessions, in a head-to-head, double-phase study with a 48-hour time interval. Twenty-seven patients underwent only ^99m^Tc-(V)DMSA and 30 patients underwent only ^99m^Tc-Sestamibi scintimammography. A total of 72/102 patients therefore underwent ^99m^Tc-(V)DMSA and a total of 75/102 patients underwent ^99m^Tc-Sestamibi scintigraphy. Inclusion and exclusion criteria for entry into the study are summarized in Table 1. The study was in accordance with the ethical principles of the Declaration of Helsinki.

Early and late planar images (at approximately 10–20 min and 60–70 min, respectively) were acquired, in the lateral prone and anterior supine positions, after intravenous administration of 925–1100 MBq radiotracer. Acquisitions were obtained using a special positioning pad (PBI-2 Scintimammography Pad Set^®^; Pinestar Technology Inc., Greenville, PA, USA).

Scintimammography was performed using a single-head γ camera (Sophycamera DS7^®^; Sopha Medical Vision International, Buc Cedex, France), equipped with a high-resolution parallel hole collimator connected to a dedicated computer (Sophy NxT^®^; Sopha Medical Vision International). The matrix was 256 × 256 pixels and the photopeak was centered at 140 keV, with a symmetrical 10% window. The acquisition time for images with both radiotracers varied between 7 min and 10 min per view (the time required to acquire 2,000,000–2,500,000 counts per image). Tomographic imaging (single-photon emission computerized tomography) was not performed since it has not been found to be superior to planar imaging, regarding the sensitivity and the specificity, for invasive breast tumors [18] and since no consensus has been reached regarding its utility. It was considered that no additional information would be obtained (except for axillary lymph node involvement detection), and tomographic imaging is time consuming and relatively inconvenient for the patient.

^99m^Tc-(V)DMSA was prepared using a domestically available kit (DMS(V)/Demoscan^®^; National Center of Physical Sciences, Institute of Radioisotopes and Radiodiagnostics 'DEMOKRITOS', Athens, Greece). The kit for the preparation of ^99m^Tc-Sestamibi (Cardiolite^®^) was obtained from Bristol-Myers Squibb GmbH (Regensburg, Germany). Both pharmaceuticals were labeled with technetium-99m within the Nuclear Medicine Department.

Diagnosis was made by histopathology of the specimens obtained surgically. The scintigraphic results and the mammograms were compared with histologic and immunohistochemical findings.

### Image analysis

Scintigrams were retrospectively evaluated, regarding the site, shape, pattern, extent, homogeneity and dispersion of radiotracer distribution. The background count rate of normal breast tissue depends on various factors (breast size, injected radioactivity dose, etc.) and is readily distinguishable from tracer accumulation in pathological tissue. Any increased focal radiotracer accumulation was assessed as suggestive of invasive breast cancer, whereas any other pattern of more widespread diffuse uptake was assessed as representing noninvasive lesions [10,15,16]. We set and evaluated as the criterion for the scintigraphic detection of noninvasive breast carcinoma the presence of any pattern of increased radiotracer accumulation other than focal activity (i.e. diffuse heterogeneous and diffuse homogeneous), independently of the coexistence of any focal increased activity. Accordingly, two experienced nuclear medicine physicians blinded to any clinical and pathologic data characterized the scintimammograms as positive or negative. Disagreement between them was resolved by consensus or by obtaining a third opinion.

Radiotracer accumulation in breast tumors was evaluated in early and delayed images (at 10–20 min and 60–70 min post injection, respectively). This was performed visually and semiquantitatively. The tumor-to-background (T/B) ratio was calculated by drawing regions of interest of standardized size and shape over the site of the greatest radioactivity within the areas of diffusely increased tracer uptake – assumed to represent noninvasive *in situ *lesions – and over the surrounding normal breast tissue. The same ratio was calculated for the areas of focal increased tracer uptake (presumably representing invasive tumor). In the late (60 min) ^99m^Tc-(V)DMSA images of some cases with extensive DCIS and associated IDC, the margins of the focal uptake area – corresponding to invasive disease – were not very clearly demarcated from the diffuse uptake. Nevertheless, the invasive component was usually clearly visible on the early (10 min) image. Thus, by comparing early and late images, that area was avoided when creating the region of interest for diffuse uptake.

### Mammography

All women underwent conventional X-ray mammography using cranio-caudal and medio-lateral projections. Images were interpreted by two experienced radiologists and were characterized as positive or negative with regard to the presence or the absence of suspicious branching and clustered coarse granular-type microcalcifications, suggestive of *in situ *carcinoma [19]. Disagreement was resolved by consensus or by obtaining a third opinion.

### Histopathology and immunostaining

The assessment of the extent of the *in situ *carcinoma was based on the number of histologic slices containing the lesion. Whole specimens were examined in serial sections and the final major diameter of the lesion was estimated by multiplying the number of slices containing the lesion by 0.3 cm, which is the medium size of a section. The EIC was defined as DCIS within and around an invasive tumor, comprising at least 25% of the neoplasm.

In order to investigate whether any of these factors is associated with the diffuse pattern of tracer distribution, an immunohistochemical method (avidin biotin peroxidase-horseradish peroxidase complex) was performed on paraffin-embedded breast tissue sections for *in situ *carcinomas, for the demonstration of Ki-67, c-*erbB-2 *and *p53 *expression. A semiquantitative estimation of these levels was performed, based on the staining intensity and the percentage of positive cells. Regarding Ki-67, a threshold of 40–45% of cells with positive staining is generally used for separating 'moderate' staining from 'intense' staining. Overexpression for c-*erbB-2 *is defined, in cases of invasive carcinoma, by dense membrane staining in ≥ 10% of the epithelial cells. Similarly, *p53 *overexpression is defined by staining in ≥ 10% of the epithelial cell nuclei. Although there is no consensus for defining c-*erbB-2 *and *p53 *overexpression in noninvasive breast carcinoma, the latter generally has the same pattern as invasive breast carcinoma and thus the same thresholds could be used [20].

### Data analysis

The T-pair test was applied to the T/B ratios of diffuse lesions between early and late acquisitions. The T/B ratios in the late (60 min) images for both tracers were also correlated with the presence of or the absence of suspicious mammographic microcalcifications, with the stromal reaction, with lymphocytic infiltration, with Ki-67 values < 40% and ≥ 40%, with c-*erbB-2 *values < 10% and ≥ 10%, and with *p53 *values < 10% and ≥ 10%, in order to assess any significant difference in diffuse tracer uptake between these populations. Finally, linear regression univariate analysis was performed to reveal any possible correlation between the T/B ratio at 60 min and the tumor size. For all tests, *P *< 0.05 was considered statistically significant.

### *In vitro *assay of ^99m^Tc-(V)DMSA uptake and micro-autoradiographic study

An *in vivo *autoradiographic study performed on tumor specimens excised immediately after scintimammography could provide precise data about ^99m^Tc-(V)DMSA localization on DCIS areas. Such a study was attempted, but the radioactivity of the tumor specimen was not sufficient. This was not surprising, since, according to the literature [21], *in vivo *autoradiographic studies in mice require approximately 37 MBq (1 mCi or 1221 MBq/kg body weight), a dose that is approximately 66 times higher than the injected dose per kilogram of body weight in humans. Given the impossibility of implementing the study as described, the tumor specimen was thus used for an *in vitro *autoradiographic study. The frozen tumor tissue of one patient with DCIS was sliced into serial sections in the cryostat microtome chamber (Microm HM 505 N^®^; Microm Laborgerate GmbH, Walldorf, Germany), was mounted onto gelatin-coated slides, was dried at 37°C for 1 hour and was then incubated in a solution containing ^99m^Tc-(V)DMSA. The sections were then exposed to Kodac X-OMATT XAR^® ^film (Eastman Kodak Co., Rochester, NY, USA) in an autoradiographic cassette for 24 hours.

## Results

Breast cancer was histologically confirmed in 46/102 women. Of these women, 26/46 had invasive carcinomas, mainly ductal (IDC) and lobular (invasive lobular carcinoma [ILC]). This was found to be the prominent histological lesion in this group of IDC patients, and these patients were therefore classified as EIC-negative. The other 20/46 cases were diagnosed as having carcinoma *in situ*. Of these, 18/20 presented DCIS: in four cases (patients 9, 12, 17, and 18; see Table 2) DCIS was the sole histological finding, while the majority (14 patients) had invasive carcinoma (IDC) associated with extensive DCIS clearly outside the confines of the invasive tumor, and thus these patients were characterized as EIC-positive. Both the remaining 2/20 cases had extensive lobular carcinoma *in situ *(LCIS), associated with an invasive component (ILC).

EIC was found more frequently in younger women (mean age ± SD, 57.1 ± 13.7 years for EIC-positive patients versus 65.8 ± 8.0 years for EIC-negative patients; *P *< 0.05). The size of the invasive tumors (IDC/ILC) associated with extensive DCIS/LCIS ranged from 0.7 to 6.0 cm (mean ± SD, 2.75 ± 1.50 cm), and the size of *in situ *carcinomas ranged from 0.8 to 9.0 cm (mean ± SD, 4.92 ± 1.93 cm). The basic data of these 20 DCIS/LCIS cases are presented on an individual basis in Table 2.

Benign breast lesions were found in the remaining 56/102 women. Among them, epithelial hyperplasia (usual type hyperplasia and/or atypical type hyperplasia) was present in 14/56 patients, with or without fibrosis, adenosis and ductal dilatation.

**Figure 1:**
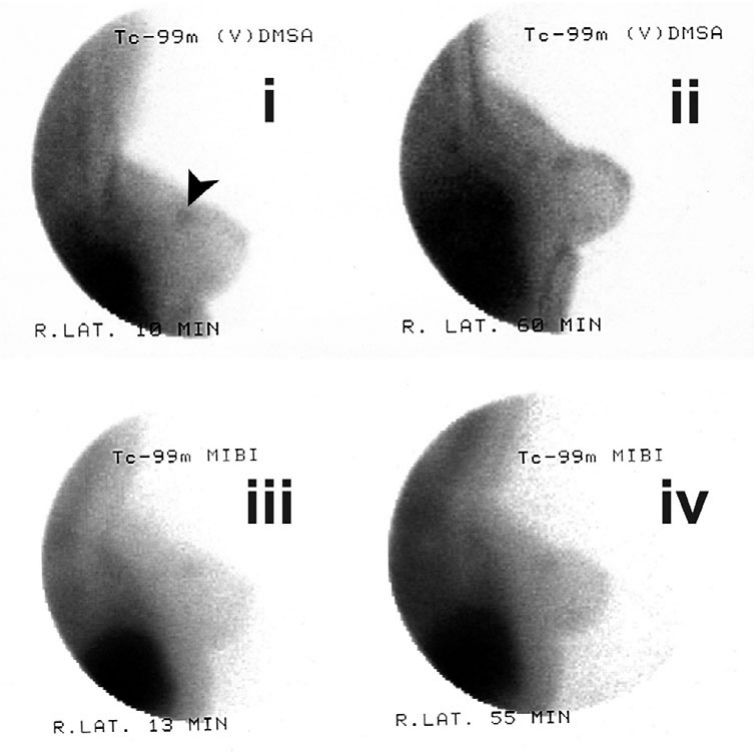
A 3-cm infiltrating ductal carcinoma of the right breast. Scintimammography, right lateral projection: ^99m^Tc-(V)DMSA at 10 min and 60 min (upper row, i-ii); ^99m^Tc-Sestamibi (^99m^Tc-MIBI) at 13 min and 55 min (bottom row, iii-iv). Focal radiotracer accumulation (arrowhead) in the upper breast hemisphere, corresponding to the invasive tumor, visible in early and late images with both radiotracers. There is no diffuse uptake pattern in the breast; the studies are considered negative for *in situ *carcinoma.

All scintimammograms were characterized as positive or negative for *in situ *carcinoma, with reference to the diagnostic criteria set for the detection of DCIS/LCIS (i.e. presence of the pattern of diffusely increased tracer uptake, regardless of the presence of any focal increased activity). Any study displaying only focal uptake was therefore a (true or false) negative for the *in situ *criterion (Fig. 1). Comparative statistics (sensitivity, specificity, accuracy, positive predictive value and negative predictive value) for each radiotracer, in the total patient groups studied with each one and in the subgroup studied with both (head-to-head study), are presented in Table 3. The interobserver agreement rate between the two physicians assessing the breast scans was 92%.

**Figure 2:**
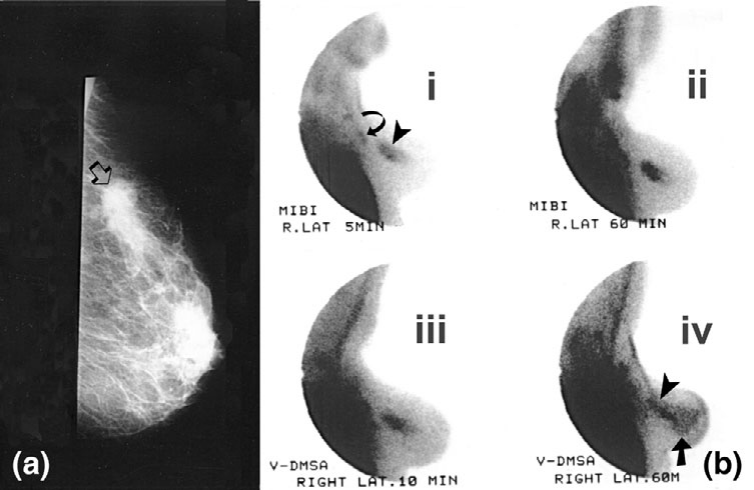
A 5-cm infiltrating ductal carcinoma, grade 2, with 9-cm, comedo-type, ductal carcinoma *in situ *of the right breast (patient 4). **(a) **Mammography, medio-lateral projection. Nodule with spiky margins (transparent arrow), close to the chest wall in the upper breast hemisphere. **(b) **Scintimammography, right lateral projection: ^99m^Tc-Sestamibi (^99m^Tc-MIBI) at 5 min and 60 min (upper row, i-ii); ^99m^Tc-(V)DMSA at 10 min and 60 min (bottom row, iii-iv). Spindle-shaped focal accumulation (arrowhead) in the area corresponding to the radiological abnormality. The 60-min ^99m^Tc-(V)DMSA image additionally reveals an extensive area of diffuse heterogeneous patchy tracer uptake (arrow) extending below and anterior to the margins of the focal accumulation. Lymph node involvement in the axilla is visible (curved arrow).

**Figure 3:**
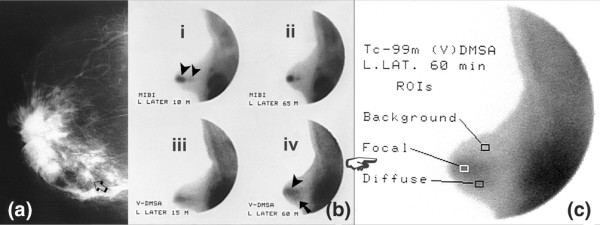
A 6-cm infiltrating ductal carcinoma, grade 2, with coexistent 5-cm ductal carcinoma *in situ*, comedo type, of the left breast (patient 1). **(a) **Mammography, medio-lateral projection. Multinodular opacity with abnormal radiating spicules and clustered microcalcifications (transparent arrow) behind the nipple. **(b) **Scintimammography, left lateral projection: ^99m^Tc-Sestamibi (^99m^Tc-MIBI) at 10 min and 65 min (upper row, i-ii); ^99m^Tc-(V)DMSA at 15 min and 60 min (bottom row, iii-iv). Increased bifocal ^99m^Tc-Sestamibi uptake (arrowheads) behind the nipple, clearly defining the invasive component of the tumor. Focal ^99m^Tc-(V)DMSA accumulation in the same area (arrowhead), with additional diffuse uptake (arrow) extending inferiorly, more prominent at 60 min and corresponding to the *in situ *tumor component. No diffuse pattern is imaged with ^99m^Tc-Sestamibi. **(c) **Scintimammography, left lateral projection: ^99m^Tc-(V)DMSA at 60 min (same as (b) iv), with regions of interest (ROIs) drawn. ROI selection for diffuse uptake with each tracer is based on the comparison between early and late images (see text).

**Figure 4:**
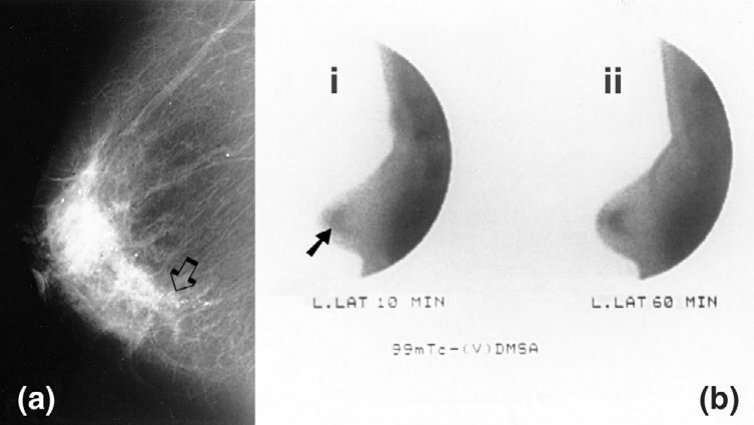
A 4.5-cm ductal carcinoma *in situ *of the left breast, comedo, solid and cribriform type (patient 18). **(a) **Mammography, medio-lateral projection. Microcalcifications (transparent arrow) behind the nipple. **(b) **Scintimammography, left lateral projection: ^99m^Tc-(V)DMSA at 10 min and 60 min (i-ii). Diffuse semi-lunar accumulation (arrow) extending behind the nipple, more prominent in the late image. ^99m^Tc-Sestamibi scan was not performed in this patient.

**Figure 5:**
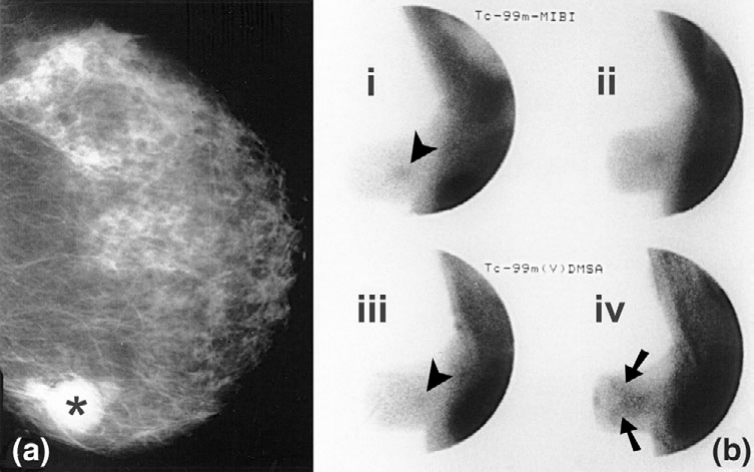
A 4-cm infiltrating lobular carcinoma, grade 3, associated with 6-cm lobular carcinoma *in situ *(LCIS) of the left breast in a 65-year-old woman (patient 20). **(a) **Mammography, cranio-caudal projection. Nodular opacity (asterisk) in the inner hemisphere of the breast. **(b) **Scintimammography, left lateral projection: ^99m^Tc-Sestamibi (^99m^Tc-MIBI) at 10 min and 60 min (upper row, i-ii); ^99m^Tc-(V)DMSA at 10 min and 60 min (bottom row, iii-iv). Focal increased uptake (arrowhead) between upper and lower breast hemisphere, imaged by both radiotracers, corresponding to an invasive tumor. A diffuse patchy 'V'-shaped tracer accumulation (arrows), surrounding the focal activity and extending anterior to it, is revealed with ^99m^Tc-(V)DMSA at 60 min only (iv). It corresponds to LCIS.

Of the 20/46 breast cancer patients with DCIS/LCIS, 19/20 underwent ^99m^Tc-(V)DMSA and 13/20 underwent ^99m^Tc-Sestamibi scintimammography (12/20 were part of the head-to-head subgroup of 45 patients that underwent both). Locally diffuse, heterogeneous (patchy), poorly circumscribed increased ^99m^Tc-(V)DMSA accumulation, sometimes covering and surrounding focal increased accumulation (if present), was noticed in 16/17 of DCIS cases (Figs 2,3,4) and in 2/2 LCIS cases (Fig. 5), a total of 18/19 (95%) patients. The one lesion that was not detected had a size of 0.8 cm (patient 9). A similar pattern of distribution was found with ^99m^Tc-Sestamibi, but only in 6/13 (46%) patients. Among those 12/20 DCIS/LCIS patients that underwent both examinations, this pattern of tracer distribution was noticed in 11/12 patients (92%) with ^99m^Tc-(V)DMSA and in 5/12 patients (42%) with ^99m^Tc-Sestamibi (Tables 3 and 4).

**Figure 6:**
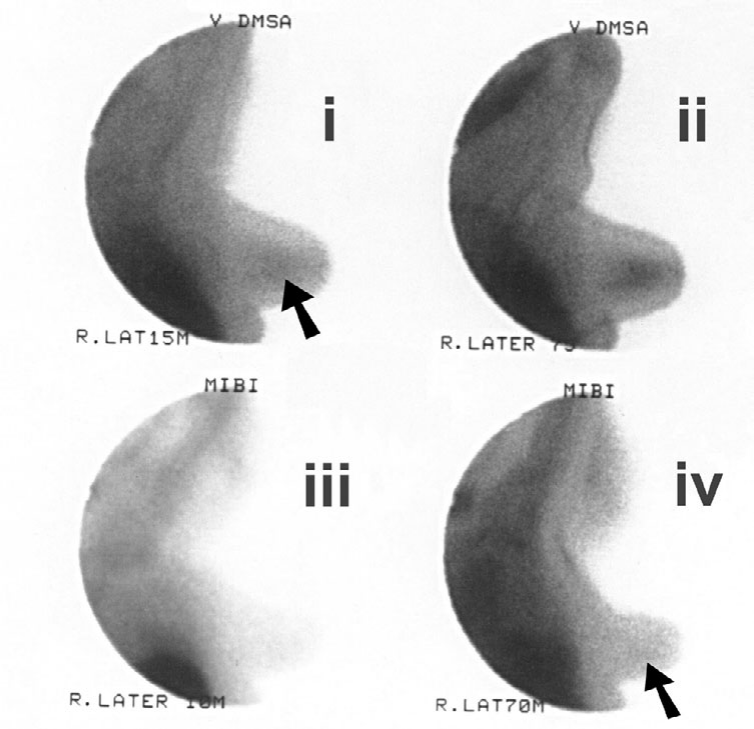
Atypical epithelial hyperplasia of the right breast in a 41-year-old woman. Scintimammography, right lateral projection: ^99m^Tc-(V)DMSA at 15 min and 75 min (upper row, i-ii); ^99m^Tc-Sestamibi (^99m^Tc-MIBI) at 10 min and 70 min (bottom row, iii-iv). Increased diffuse homogeneous ^99m^Tc-(V)DMSA uptake (arrow) at early and late acquisitions, more prominent in the late image. Very faint (hardly visible) ^99m^Tc-Sestamibi activity (arrow) in the same area.

In women with histologically confirmed usual type hyperplasia and/or atypical type hyperplasia, a similar pattern of intense diffuse, not patchy, but more homogeneous, ^99m^Tc-(V)DMSA distribution was observed in 10/14 patients (71%). Diffuse accumulation of ^99m^Tc-Sestamibi in sites of epithelial hyperplasia was observed in 6/14 patients (43%), yet it was less intense in comparison with ^99m^Tc-(V)DMSA (Fig. 6). In the absence of epithelial hyperplasia, the benign histological lesions of fibrosis, adenosis and ductal dilatation alone did not accumulate either of the two radiotracers. These results are also presented in Table 4.

In the series studied, the T/B ratio for ^99m^Tc-(V)DMSA uptake in the sites of diffuse accumulation pattern demonstrated a tendency to increase over time (Figs 2,3,4,5,6). Semiquantitative analysis (T/B ratio) performed separately in DCIS and in IDC revealed a statistically significant increase in late images only of the diffuse ^99m^Tc-(V)DMSA accumulation in DCIS (mean ± SD, 1.27 ± 0.22 at 10 min versus 1.76 ± 0.25 at 60 min; *P *< 0.01). On the contrary, T/B ratios for the focal ^99m^Tc-(V)DMSA uptake in invasive tumors were not significantly increased in the late acquisitions (mean ± SD, 1.76 ± 0.28 at 10 min versus 1.94 ± 0.28 at 60 min; *P *> 0.05). The pattern of diffuse ^99m^Tc-(V)DMSA distribution observed in epithelial hyperplasia demonstrated a similar tendency to increase over time. The T/B ratios for ^99m^Tc-Sestamibi did not demonstrate significant variability with time, either in DCIS or in IDC (mean ± SD for DCIS, 1.34 ± 0.29 at 10 min versus 1.33 ± 0.33 at 60 min; *P *> 0.05; and mean ± SD for IDC, 2.06 ± 0.97 at 10 min versus 1.98 ± 0.84 at 60 min; *P *> 0.05). Similarly, the diffuse ^99m^Tc-Sestamibi distribution observed in epithelial hyperplasia did not appear to increase significantly over time. Patients with LCIS and associated ILC gave parallel results, but due to the limited number of cases (*n *= 2) they could not be statistically evaluated.

Linear regression univariate analysis revealed no statistically significant correlation between the ^99m^Tc-(V)DMSA T/B ratio at 60 min and tumor size (*r *= 0.28, *P *> 0.1).

Mammography depicted suspicious microcalcifications in 10/20 (50%) patients with DCIS/LCIS (Table 2). The diffuse uptake of ^99m^Tc-(V)DMSA in DCIS/LCIS at 60 min was significantly higher in patients with suspicious microcalcifications on mammography as compared with patients without (mean ± SD T/B ratio, 1.81 ± 0.05 versus 1.4 ± 0.07, respectively; *P *= 0.003). ^99m^Tc-Sestamibi diffuse uptake at 60 min was not significantly different between the patient groups with and without microcalcifications (mean ± SD T/B ratio, 1.49 ± 0.19 versus 1.31 ± 0.09, respectively; *P *= 0.49).

An intense stromal reaction was found in 1/20 patient, a moderate stromal reaction in 4/20 patients, and a mild stromal reaction in 3/20 patients with DCIS/LCIS; a total of 8/20 patients (40%). Microcalcifications combined with stromal reaction appeared in 6/20 cases. Mild or moderate lymphocytic infiltration was noted in 4/20 patients (20%). Pure comedo was the most prominent type (10/18) among DCIS (Table 2). No statistically significant difference was found in ^99m^Tc-(V)DMSA or ^99m^Tc-Sestamibi uptake between patients with stromal reaction and/or lymphocytic infiltration and those patients without.

With reference to the studied immunohistochemical parameters in DCIS/LCIS patients, the Ki-67 value ranged from 10% to 55% (mean ± SD, 33.4 ± 13.4%), *p53 *ranged from 0% to 27% (mean ± SD, 6.4 ± 8.8%) and c-*erbB-2 *ranged from 0% to 80% (mean ± SD, 21.7 ± 26.4%) (Table 2).

The uptake of ^99m^Tc-(V)DMSA was significantly higher in patients with Ki-67 overexpression (≥ 40%) as compared with patients with Ki-67 levels < 40% (mean ± SD T/B ratio at 60 min, 1.86 ± 0.027 versus 1.52 ± 0.047, respectively; *P *= 0.025]. No significant difference was found in ^99m^Tc-Sestamibi scans in relation to Ki-67 levels ≥ 40% and < 40% (mean ± SD T/B ratio at 60 min, 1.51 ± 0.27 versus 1.24 ± 0.08, respectively; *P *= 0.46).

Overexpression of c-*erbB-2 *(≥ 10%) was associated with higher ^99m^Tc-(V)DMSA uptake in DCIS/LCIS (mean ± SD T/B ratio at 60 min, 1.89 ± 0.01 versus 1.52 ± 0.09 for values ≥ 10% and < 10%, respectively; *P *= 0.004). ^99m^Tc-Sestamibi uptake was not significantly different between women with c-*erbB-2 *values ≥ 10% and < 10% (mean ± SD T/B ratio at 60 min, 1.43 ± 0.06 versus 1.3 ± 0.16, respectively; *P *= 0.64).

Diffuse uptake of both ^99m^Tc-(V)DMSA and ^99m^Tc-Sestamibi was not significantly different with regard to *p53 *values < 10% and ≥ 10% (mean ± SD T/B ratio at 60 min, 1.71 ± 0.068 versus 1.72 ± 0.06, respectively, for ^99m^Tc-(V)DMSA; *P *= 0.93; and 1.60 ± 0.11 versus 1.29 ± 0.17, respectively, for ^99m^Tc-Sestamibi; *P *= 0.78).

The c-*erbB-2 *levels were significantly higher in DCIS cases with coexistent IDC (EIC-positive) than in EIC-negative patients (*P *< 0.05), whereas *p53 *expression did not demonstrate such a statistically significant difference.

**Figure 7:**
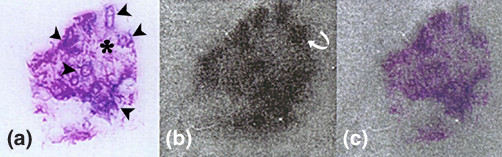
Ductal carcinoma *in situ *(DCIS), comedo, solid and cribriform type (patient 18). **(a) **Tumor section (hematoxylin & eosin, × 25). Regions of DCIS (arrowheads), within normal breast tissue (asterisk). **(b) ***In vitro *^99m^Tc-(V)DMSA autoradiogram of the same section (× 25). Distribution of the radioactivity in the same tumor section. The sites of intense tracer uptake (curved arrow) appear darker. **(c) **Overlay of stained tumor section and autoradiogram. The histologically detected lesion is well correlated with the tissue sites of intense ^99m^Tc-(V)DMSA uptake.

*In vitro *autoradiography of the breast tumor of one patient with DCIS revealed a very good correlation between the regional distribution of ^99m^Tc-(V)DMSA radioactivity and the distribution of DCIS cell clumps, as observed after hematoxylin & eosin staining of the same sections (Fig. 7).

## Discussion

*In situ *breast carcinoma tumors are very difficult to identify, and the only available method to date for their detection is the evaluation of the shape and distribution of microcalcifications on mammography, with all the limitations inherent in this method (dense breasts, scars, implants, etc.).

Scintigraphic imaging of EIC, DCIS or epithelial hyperplasia with ^99m^Tc-(V)DMSA has already been reported [15,16]. Few reports have been published concerning the ability of ^99m^Tc-Sestamibi not actually to detect DCIS, but rather to improve invasive breast cancer detection in the presence of DCIS [9,10], and these studies seem to be contradictory. Howarth and colleagues [10] reported no change in sensitivity in the presence or absence of carcinoma *in situ*, and furthermore that there is no relationship between ^99m^Tc-Sestamibi uptake and DCIS, except in the presence of an invasive tumor. Several other authors, however, have suggested that diffuse ^99m^Tc-Sestamibi uptake in benign breast disorders was associated with proliferative changes [15,22,23], demonstrating an increased risk of developing into breast cancer, while other workers described hormonal influence to be the cause of diffuse breast uptake on scintimammography [24]. In the present study, hormonal influence or the menstrual cycle could not be responsible for this kind of tracer distribution, since all but two women were postmenopausal and they were not on any hormone replacement therapy.

In the group of patients with breast malignancy, the detected invasive tumors (IDC/ILC) presented mostly with focal increased uptake of both radiotracers. On the contrary, the pattern of diffuse heterogeneous (patchy) uptake was mainly observed in cases with DCIS, independently of the presence of an invasive tumor. Among the 26 invasive tumors not associated with extensive amounts of *in situ *carcinoma, only a couple of cases gave false positive results (for DCIS) with ^99m^Tc-(V)DMSA (Table 4): one consisted of multiple microscopic foci of IDC, and the other case presented IDC with intense lymphocytic infiltration.

LCIS was found in two cases, and due to this limited number specific scintigraphic results could not be statistically evaluated. They could have been considered as false positive findings for DCIS, yet their scintigraphic appearance was the same; they both revealed a similar diffuse inhomogeneous pattern of ^99m^Tc-(V)DMSA uptake, apart from the focal uptake corresponding to the coexistent ILC. They could therefore not be separated from DCIS, except after histological examination of the biopsied tissue, and thus they are studied together in this series. If this diffuse uptake was attributable to the lobular invasive component, known for its peculiar pattern of growth and local invasion, then it should have been clearly visible from the early (10 min) images, simultaneously with the focal uptake. Yet the extensive diffuse ^99m^Tc-(V)DMSA uptake was prominent in the late (60 min) image, whereas the focal tracer uptake was also visible in the early image (Fig. 4). ^99m^Tc-Sestamibi, known for its efficacy in imaging invasive carcinoma, in this case managed to image only the focal uptake in both early and late images.

The diffuse pattern of radiotracer uptake (not patchy, but more homogeneous) was also noticed in some cases of epithelial hyperplasia, more prominently with ^99m^Tc-(V)DMSA. Epithelial hyperplasia was therefore the major source of false positive results. The discrimination between atypical epithelial hyperplasia and *in situ *carcinoma is certainly not easy, even on frozen section. Yet any false positive scan attributable to hyperplasia should not be considered a major disadvantage, since it may lead to a search but not to an incorrect final decision concerning excision. This is because scintigraphic data (in addition to suspicious microcalcifications, if present) can serve as a guide and provide the surgeon with useful information preoperatively, in order to decide the extent of the search and to facilitate a guided biopsy, rather than directly determine whether an excision has to be performed.

Increased cell proliferation activity (Ki-67 ≥ 40%) was found to be significantly correlated with the diffuse ^99m^Tc-(V)DMSA uptake in breast carcinoma *in situ*. Papantoniou and colleagues [25] recently reported this factor to be independently correlated with focal ^99m^Tc-(V)DMSA accumulation in invasive breast cancer, while their preliminary reports [26] revealed that cases of usual type hyperplasia imaged with ^99m^Tc-(V)DMSA tend to be related to elevated Ki-67 values and are therefore at risk of developing breast malignancy, according to the literature [27-31]. Cutrone and colleagues claimed that increased proliferative activity is found not only in invasive cancers, but also in precancerous lesions [27].

Shaaban and colleagues [28], along with several other reports [29,30], suggested that benign breast lesions such as epithelial hyperplasia, when associated with increased levels of Ki-67 and estrogen receptors type A, define a subset of hyperplastic lesions with high risk of subsequent breast cancer development. Such patients could be selected for prophylactic antiestrogen therapy to diminish the proliferative activity [28,31]. The observed tendency of the ^99m^Tc-(V)DMSA diffuse uptake pattern to increase over time (being more notable on the delayed 60-min images), as well as its relation to elevated proliferative cellular activity (Ki-67), could be considered indicative of the existence of a distinct primary pathway for the mechanism of its accumulation in tumor tissue. This is considered to reflect the tracer's uptake in structures that participate in mitotic activity of cancerous and precancerous cell populations [25]. The possibility for this finding to be due to locally increased vascularity, angiogenesis or vessel permeability is therefore highly unlikely; if that was the case, then this diffuse pattern would have been equally clearly visible in the early 10-min images.

On the contrary, diffuse ^99m^Tc-Sestamibi distribution was not found to be significantly correlated with Ki-67 expression. Palmedo and colleagues [32] reported that the ^99m^Tc-Sestamibi concentration was one-half that of ^99m^Tc-(V)DMSA in an experimental model of rats bearing poorly differentiated breast tumours. Cutrone and colleagues found a moderate correlation between ^99m^Tc-Sestamibi uptake and the degree of cellular proliferation [27], while Cwikla and colleagues did not [33]. Angiogenesis and an oxidative metabolism seem to be favorable factors for ^99m^Tc-Sestamibi tumor uptake, rather than proliferative activity [25,29]. This could provide an explanation for the lower sensitivity of this radiotracer, as compared with ^99m^Tc-(V)DMSA, in imaging such diffuse lesions.

Overexpression of c-*erbB-2 *(≥ 10%) is found in almost 60% of cases of high-grade comedo-type DCIS, in 10–40% of IDC and in only a few cases of ILC [34,35]. An increased c-*erbB-2 *level was a factor significantly correlated with diffuse ^99m^Tc-(V)DMSA uptake (but not with ^99m^Tc-Sestamibi uptake). This appears reasonable, since oncoprotein overexpression is usually found in aggressive *in situ *carcinomas with increased levels of cell proliferative activity.

The measurement of Ki-67 and c-*erbB-2 *on histological specimens is not related to the size of the tumor. In the present series, the T/B ratio for ^99m^Tc-(V)DMSA in the late image was not found to be significantly correlated with tumor size. It is therefore considered that the correlation found for the ^99m^Tc-(V)DMSA T/B ratio to Ki-67 and/or c-*erbB-2 *expression is independent of tumor size.

*p53 *overexpression is known to reflect tumor aggressiveness and a decreased disease-free interval following therapy. An intriguing finding requiring further investigation, however, is that *p53 *overexpression, unlike that of Ki-67 and c-*erbB-2*, was not significantly correlated with this pattern of uptake of either tracer in the present series.

DCIS of the comedo type is usually accompanied by a desmoplastic stromal reaction with pronounced neovascularization. Elastosis is also more common in DCIS [36]. A variable inflammatory infiltrate is present in the periductal stroma. This consists of lymphocytes and histiocytes, in amounts ranging from sparse to abundant [37]. The suggestion that stromal reaction and/or lymphocytic infiltration could be considered mediators for the diffuse radiotracer uptake was not confirmed in the studied population.

Suspicious mammographic microcalcifications were observed in only one-half of the DCIS/LCIS cases, whereas ^99m^Tc-(V)DMSA revealed a diffuse uptake pattern in the vast majority (18/19 patients). Nevertheless, diffuse uptake for ^99m^Tc-(V)DMSA (but not for ^99m^Tc-Sestamibi) was found to be significantly increased in women with suspicious microcalcifications as compared with those without. A possible explanation for this could be that clustered microcalcifications are usually found in more aggressive types of DCIS (such as comedo type), which tend to be related to increased Ki-67 levels. The majority of DCIS found in this series were of comedo type. Although it has been reported for the comedo type that suspicious microcalcifications approximately correspond to the histologically confirmed size, their extent in the present study was substantially smaller in comparison with the spread of diffuse ^99m^Tc-(V)DMSA uptake, which in turn was found to correlate very well with the histologically confirmed size of DCIS. These findings imply that calcium deposits may not represent a ^99m^Tc-(V)DMSA uptake mechanism, as was previously presumed [16].

Examining scintimammograms in combination with mammography could help limit false positive results. In the presence of suspicious microcalcifications, a diffuse ^99m^Tc-(V)DMSA uptake could be considered more suggestive of *in situ *carcinoma. In the absence of microcalcifications, however, a diffuse ^99m^Tc-(V)DMSA uptake could imply the possibility of a false positive study, probably due to epithelial hyperplasia.

*In vitro *^99m^Tc-(V)DMSA autoradiography of one tumor specimen demonstrated a very good correlation between radioactivity uptake and the distribution of foci of DCIS cells. This is considered a strong indication for the tracer localization in these cell clumps. Autoradiography nevertheless needs to be performed in a larger series of tumor specimens to verify the findings.

## Conclusion

^99m^Tc-(V)DMSA displayed an excellent sensitivity and negative predictive value in detecting DCIS/LCIS, especially in cases associated with increased cell proliferation (Ki-67) and c-*erbB-2 *overexpression, independent of the presence of suspicious microcalcifications. Its relatively lower specificity and positive predictive value, caused by false positive scans mainly attributable to epithelial hyperplasia, should not be considered a major disadvantage, since to date there is no diagnostic technique other than suspicious mammographic microcalcifications to define high-risk breast areas that should be biopsied. ^99m^Tc-Sestamibi appeared to be less sensitive, yet it demonstrated a very good specificity and could also be useful in preinvasive lesion imaging.

In our opinion, any diffuse tracer accumulation, either as an isolated finding or extending beyond the margins of a focal, well-circumscribed accumulation, could be considered to probably correspond with highly proliferating tissue and to possibly represent DCIS/LCIS or epithelial hyperplasia. Since the difference between *in situ *carcinoma and atypical type hyperplasia cannot always be discriminated, even histologically, we note the potential usefulness of scintimammography in imaging these lesions and making them accessible to biopsy. There is, however, a need for a larger series of patients to verify these preliminary observations, as well as prospective studies to estimate its reliability in affecting treatment decisions.

## Abbreviations

DCIS = ductal carcinoma *in situ*; EIC = extensive intraductal carcinoma; IDC = invasive ductal carcinoma; ILC = invasive lobular carcinoma; Ki-67 = cell proliferation index; LCIS = lobular carcinoma *in situ*; SD = standard deviation; T/B, tumor-to-background; ^99m^Tc-(V)DMSA = technetium-99m pentavalent dimercaptosuccinic acid; ^99m^Tc-Sestamibi (^99m^Tc-MIBI) = technetium-99m 2-methoxy isobutyl isonitrile.

## Competing interests

The author(s) declare that they have no competing interests.

## Authors' contributions

VP is the guarantor of integrity of the entire study and was responsible for study concepts, study design, literature research, data acquisition, data analysis/interpretation, statistical analysis, manuscript preparation, manuscript definition of intellectual content, manuscript editing, and manuscript revision/review. ST participated in data acquisition, data analysis/interpretation, statistical analysis, manuscript preparation, manuscript editing, and manuscript revision/review. EM, VV, MSou, LN, and AT participated in data acquisition, data analysis/interpretation, and manuscript preparation. MSot and IP participated in experimental studies (autoradiography *in vitro*), data acquisition, data analysis/interpretation, and manuscript preparation. DL, AL, and MM participated in clinical studies, data acquisition, data analysis/interpretation, and manuscript preparation. JK participated in data acquisition, data analysis/interpretation, statistical analysis, and manuscript preparation. CZ participated in data analysis/interpretation, manuscript preparation, manuscript definition of intellectual content, manuscript editing, and manuscript revision/review. All authors read and approved the final manuscript.

## Figures and Tables

**Table 1:** Inclusion and exclusion criteria of the study

Inclusion criteria	Exclusion criteria
Female sex	Pregnancy
Age older than 21 years	Recurrent disease
Not pregnant	Previous mastectomy
Suspicious lesion of the breast (on palpation or mammography)	Fine needle aspiration within 1 week prior to scintimammography
Recommendation for excisional biopsy after mammography	Core biopsy during the previous 4 weeks
Informed consent of the patient	Previous chemotherapy
	Medically unstable patient (severe arrhythmia, heart failure or recent surgery)

**Table 2:** Data of the 20 patients diagnosed with ductal carcinoma *in situ*/lobular carcinoma *in situ *(DCIS/LCIS)

Patient	Age (years)	Histology	Grade of invasive carcinoma (IDC/ILC)	Size of IDC/ILC (cm)	Type of DCIS/LCIS	Size of DCIS/LCIS (cm)	Mammography with suspicious microcalcifications	Scintimammography				Stromal reaction	Lymphocytic infiltration	*p53 *(%)	c-*erb B-2 *(%)	Ki-67 (%)
								^99m^Tc-(V) DMSA		^99m^Tc-Sestamibi						
													
								Focal uptake	Diffuse uptake	Focal uptake	Diffuse uptake					
1	50	IDC + DCIS	II	6.0	Comedo	5.0	(+)	(+)	(+)	(+)	(+)	-	-	8	8	50
2	58	IDC + DCIS	III	2.5	Comedo	5.0	(-)	(+)	(+)	(+)	(-)	-	-	0	0	25
3	49	IDC + DCIS	I	1.0	Comedo	6.0	(+)	(-)	(+)	(-)	(-)	++	-	0	20	55
4	65	IDC + DCIS	II	5.0	Comedo	9.0	(-)	(+)	(+)	(+)	(+)	-	-	10	50	35
5	54	IDC + DCIS	II	5.0	Comedo	7.0	(+)	(+)	(+)	(+)	(+)	-	-	0	50	45
6	48	IDC + DCIS	II	2.5	Solid	5.0	(-)	(+)	(+)	(-)	(-)	++	+	0	0	15
7	77	IDC + DCIS	III	3.0	Comedo + cribriform	4.0	(+)	(+)	(+)	(+)	(-)	+	-	0	0	35
8	40	IDC + DCIS	II	1.5/1.8/2.0 (multifocal)	Comedo	6.0	(+)	(+)	(+)	(+)	(-)	+	-	10	10	10
9	50	DCIS			Comedo	0.8	(-)	(-)	(-)	(-)	(-)	-	-	8	15	32
10	53	IDC + DCIS	II	1.5	Comedo	4.0	(-)	(+)	(+)	(+)	(+)	-	-	25	0	35
11	86	IDC + DCIS	III	2.0	Comedo	3.0	(-)	(+)	(+)	Not performed		-	-	0	0	42
12	66	DCIS			-	3.0	(-)	(-)	(+)	Not performed		-	-	0	0	35
13	71	IDC + DCIS	II	0.7	Cribriform	8.0	(+)	(+)	(+)	Not performed		+++	+	0	80	55
14	60	IDC + DCIS	II	2.9	Comedo + micropapillary	5.0	(-)	(+)	(+)	(+)	(+)	-	-	27	75	15
15	50	IDC + DCIS	II	2.0	Micropapillary	2.0	(-)	Not performed		(+)	(+)	-	-	0	0	31
16	38	IDC + DCIS	III	4.3	Comedo	4.0	(+)	(+)	(+)	Not performed		+	++	0	50	45
17	61	DCIS			Comedo + solid	5.0	(+)	(-)	(+)	Not performed		-	-	20	10	15
18	71	DCIS			Comedo + solid + cribriform	4.5	(+)	(-)	(+)	Not performed		-	-	13	40	35
19	67	ILC + LCIS	II	2.5		6.0	(+)	(+)	(+)	Not performed		++	-	0	0	20
20	65	ILC + LCIS	III	4.0		6.0	(-)	(+)	(+)	(+)	(-)	++	++	6	25	38

Patients 1–10, patient 14 and patient 20 belong to the 45 patients of the head-to-head subgroup that underwent both scintigraphic examinations. IDC, invasive ductal carcinoma; ILC, invasive lobular carcinoma; ^99m^Tc-(V)DMSA, technetium-99m pentavalent dimercaptosuccinic acid; ^99m^Tc-Sestamibi (^99m^Tc-MIBI), technetium-99m 2-methoxy isobutyl isonitrile; (+), positive scan or mammogram; (-), negative scan or mammogram; -, absent stromal reaction or lymphocytic infiltration; +, mild stromal reaction or lymphocytic infiltration; ++, moderate stromal reaction or lymphocytic infiltration; +++, intense stromal reaction or lymphocytic infiltration.

**Table 3:** Statistics for each radiotracer concerning *in situ *carcinoma detection, in the total group of patients studied and in the subgroup that underwent both examinations (double phase study)

	^99m^Tc-(V)DMSA radiotracer		^99m^Tc-Sestamibi radiotracer	
	
	Head-to-head patients (45/102)	Total patients (72/102)	Head-to-head patients (45/102)	Total patients (75/102)
Sensitivity	92%	95%	42%	46%
Specificity	91%	77%	94%	90%
Accuracy	91%	82%	80%	83%
Positive predictive value	79%	60%	71%	50%
Negative predictive value	97%	98%	82%	89%

^99m^Tc-(V)DMSA, technetium-99m pentavalent dimercaptosuccinic acid; ^99m^Tc-Sestamibi (^99m^Tc-MIBI), technetium-99m 2-methoxy isobutyl isonitrile.

**Table 4:** Scintigraphic identification of breast carcinoma *in situ *with ^99m^Tc-(V)DMSA and ^99m^Tc-Sestamibi: classification of the studied population (*n *= 102) according to the histological diagnosis and the scintimammographic criterion set for ductal carcinoma *in situ*/lobular carcinoma *in situ *(DCIS/LCIS)

	Malignant lesions (*n *= 46)								Benign lesions (*n *= 56)							
Histology	Carcinoma *in situ*, alone, or with invasive component (DCIS/LCIS ± IDC/ILC) (*n *= 20)				Invasive carcinoma (IDC/ILC) (*n *= 26)				Lesions with usual type, or atypical hyperplasia (UTH/ATH) (*n *= 14)				Lesions without hyperplasia (fibrosis, adenosis and ductal dilatation) (*n *= 42)			
Radiotracer	^99m^Tc-(V)DMSA (*n *= 19)		^99m^Tc-Sestamibi (*n *= 13)		^99m^Tc-(V)DMSA (*n *= 25)		^99m^Tc-Sestamibi (*n *= 20)		^99m^Tc-(V)DMSA (*n *= 14)		^99m^Tc-Sestamibi (*n *= 14)		^99m^Tc-(V)DMSA (*n *= 14)		^99m^Tc-Sestamibi (*n *= 28)	
Results (-) (*n *= 28)	(+) (*n *= 18)	(-) (*n *= 1)	(+) (*n *= 6)		(-) (*n *= 7)	(+) (*n *= 2)	(-) (*n *= 23)	(+) (*n *= 0)	(-) (*n *= 20)	(+) (*n *= 10)	(-) (*n *= 4)	(+) (*n *= 6)	(-) (*n *= 8)	(+) (*n *= 0)	(-) (*n *= 14)	(+) (*n *= 0)
Classification	TP	FN	TP	FN	FP	TN	FP	TN	FP	TN	FP	TN	FP	TN	FP	TN

^99m^Tc-(V)DMSA, technetium-99m pentavalent dimercaptosuccinic acid; ^99m^Tc-Sestamibi (^99m^Tc-MIBI), technetium-99m 2-methoxy isobutyl isonitrile; EIC, extensive intraductal component; IDC, invasive ductal carcinoma; ILC, invasive lobular carcinoma; UTH, usual type hyperplasia; ATH, atypical type hyperplasia; (+), positive scan; (-), negative scan; TP, true positive; FN, false negative; FP, false positive; TN, true negative.
